# Clinical value of serum JKAP in acute ischemic stroke patients

**DOI:** 10.1002/jcla.24270

**Published:** 2022-03-10

**Authors:** Jianli Zhang, Jing Yang, Jingchun Hu, Weiwei Zhao

**Affiliations:** ^1^ Department of Neurology Lishui Municipal Central Hospital Lishui Hospital of Zhejiang University The Fifth Affiliated Hospital of Wenzhou Medical University Lishui China; ^2^ Department of Endocrinology Zhuji People’s Hospital of Zhejiang Province Zhuji China; ^3^ Department of Anesthesiology Lishui Municipal Central Hospital Lishui Hospital of Zhejiang University The Fifth Affiliated Hospital of Wenzhou Medical University Lishui China; ^4^ Department of Rehabilitation The First Hospital of Jiaxing Affiliated Hospital of Jiaxing University Jiaxing China

**Keywords:** acute ischemic stroke, disease severity, inflammation cytokines, Jun N‐terminal kinase pathway‐associated phosphatase, prognosis

## Abstract

**Background:**

Jun N‐terminal kinase pathway‐associated phosphatase (JKAP) regulates neuronal function, T helper (Th) 1/2/17 cell differentiation, and inflammatory process, but its clinical role in acute ischemic stroke (AIS) patients remains unclear. Hence, this study intended to evaluate JKAP level and its relationship with disease severity, Th1, 2, 17 secreted cytokines, adhesion molecules, and prognosis of AIS patients.

**Methods:**

Serum JKAP of 122 AIS patients and 50 controls was detected by ELISA. For AIS patients only, Th1, 2, 17 secreted cytokines IFN‐γ, IL‐4, IL‐17; TNF‐α, ICAM‐1, and VCAM‐1 were also detected by ELISA.

**Results:**

JKAP was decreased in AIS patients compared with controls (46.350 (interquartile range (IQR): 34.250–59.875) pg/ml vs. 84.500 (IQR: 63.175–113.275) pg/ml, *p* < 0.001), which could distinguish AIS patients from controls (area under curve (AUC): 0.810, 95% confidence interval (CI): 0.732–0.888). In AIS patients, JKAP negatively linked with the National Institutes of Health Stroke Scale (NIHSS) score (*r_s_* = −0.342, *p* < 0.001); besides, it was positively related to IL‐4 (*r_s_* = 0.213, *p* = 0.018) and negatively associated with IL‐17 (*r_s_* = −0.270, *p* = 0.003) but not related to IFN‐γ (*r_s_* = −0.146, *p* = 0.109). Furthermore, elevated JKAP associated with declined TNF‐α (*r_s_* = −0.219, *p* = 0.015) and ICAM‐1 (*r_s_* = −0.235, *p* = 0.009) but not related to VCAM‐1 (*r_s_* = −0.156, *p* = 0.085). Besides, declined JKAP was linked with 2‐year recurrence (*p* = 0.027) and 3‐year recurrence (*p* = 0.010) in AIS patients; while JKAP was not related to 1‐year recurrence or death risk (both *p* > 0.050).

**Conclusion:**

JKAP may sever as a candidate prognostic biomarker in AIS patients, indicating its potency for AIS management.

## Graphical abstract

This study detected JKAP, IFN‐γ, IL‐4, IL‐17, TNF‐α, ICAM‐1, and VCAM‐1 in 122 AIS patients and JKAP in 50 controls to evaluate the association of JKAP with disease severity, Th1, 2, 17 secreted cytokines, adhesion molecules, and prognosis of AIS patients. Interestingly, it was found that JKAP was decreased in AIS patients than controls. Besides, JKAP negatively correlated with NIHSS score, IL‐17, TNF‐α, and ICAM‐1 in AIS patients, while it was positively related to IL‐4. Additionally, declined JKAP was linked with 2‐year recurrence and 3‐year recurrence in AIS patients.

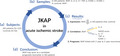

## 1 INTRODUCTION

Acute ischemic stroke (AIS), also called cerebrovascular accident, is an acute onset of focal neurological deficits caused by cerebral vascular occlusion.^1^, ^2^, ^3^, ^4^ According to the 2021 heart disease and stroke statistics, it is estimated that 795,000 people in the United States encounter stroke each year and 87% of them are AIS patients.^5^ Aiming to minimize stroke size, optimize recovery, and avoid comorbidities, diverse treatments (including thrombolysis, antiplatelet therapy, anticoagulants, etc.) have been applied.^6^, ^7^ However, the management of AIS is still a large challenge with high recurrence rate (1‐year recurrence rate ranging from 7% to 20%), fatality rate (around 21% 1‐month death rate), and disability rate (approximately 36%–71%).^5^, ^8^, ^9^, ^10^ Hence, aiming to improve the management of AIS, it is meaningful to explore biomarkers which could help clinicians identify high‐risk AIS patients and monitor their prognosis.

Jun N‐terminal kinase pathway associated phosphatase (JKAP), also named dual‐specificity phosphatase 22 (DUSP22), is a tyrosine‐specific protein that dephosphorylates mitogen‐activated protein (MAP) kinase.^11^, ^12^, ^13^, ^14^ According to previous studies, JKAP not only regulates neuronal function but also moderates immune and inflammatory process in several neurological and inflammation‐mediated diseases (including Parkinson's disease, Alzheimer's disease, sepsis, etc.).^15^, ^16^, ^17^ For instance, one study finds that JKAP inhibits CD4^+^ T‐cell activation as well as its differentiation into T helper (Th) 1 and Th17 cells in Parkinson's disease patients, but JKAP is not related to Th2 cells.^15^ Besides, JKAP also associates with stenosis of blood vessels and involves in ameliorating the neointimal hyperplasia induced by cessation of blood flow.^18^, ^19^ Combining above aspects, we speculated that JKAP might participate in AIS pathogenesis and have potential to work as a biomarker in AIS patients, while there is no clinical study reporting relevant findings.

Therefore, this study detected serum JKAP level in 122 AIS patients and 50 controls, aiming to evaluate its correlation with disease severity, Th1, 2, 17 secreted cytokines, adhesion molecules, and prognosis of AIS patients.

## 2 METHODS

### 2.1 Participants

From January 2017 to January 2018, 122 newly diagnosed AIS patients were serially included in this study. The inclusion criteria were as follows: (i) newly confirmed as AIS in accordance with the guideline issued by American Stroke Association (ASA)^20^; (ii) aged over 18 years; (iii) absent of intracranial hemorrhage; (iv) willing to provide peripheral blood (PB) samples; (v) willing to be followed up regularly. The patients conforming to the following conditions were excluded from the study: (i) complicated with severe infection, inflammatory disease or immune system disease; (ii) had history of solid tumor or hematological disease. In addition, between January 2017 and January 2018, the study also included 50 subjects with at least 2 of the high‐risk factors (smoke, hypertension, hyperlipidemia, hyperuricemia, diabetes mellitus, and chronic kidney disease (CKD)) as controls. For study analysis, the age range and gender ratio of controls were matched to those of AIS patients: age range, 50–80 years old; gender ratio, 3:2 (male vs. female). The controls who had a prior history of stroke were ineligible for the study, and the exclusion conditions for AIS patients were also appropriate for the controls. The study was approved by the Ethics Committee. All subjects provided the written informed consents.

### 2.2 Data document and sample preparation

Demographics and underlying diseases of all subjects were documented for study analysis, and the National Institutes of Health Stroke Scale (NIHSS) score of AIS patients were obtained for the assessment of disease severity. Additionally, PB samples were collected from all AIS patients after admission, as well as from all controls after recruitment, then serum was isolated for the further detections.

### 2.3 Sample assessment

All collected serum samples were applied to evaluate JKAP level by enzyme‐linked immunosorbent assay (ELISA) using commercial JKAP Human ELISA Kits (Shanghai Enzyme‐linked Biotechnology Co., Ltd). Besides, the serum samples of AIS patients were also applied to assess the level of interferon gamma (IFN‐γ), interleukin‐4 (IL‐4), interleukin‐17 (IL‐17), tumor necrosis factor alpha (TNF‐α), intercellular adhesion molecule 1 (ICAM‐1), and vascular cell adhesion molecule 1 (VCAM‐1) by ELISA using commercial Human ELISA Kits (Bio‐Techne China Co., Ltd.). The procedures of ELISA were in strict accordance with the instructions provided by manufacturers.

### 2.4 Thrombolysis treatment

For AIS patients within 3 h of onset, intravenous thrombolytic therapy was implemented; for AIS patients within 6 h of onset, arterial thrombectomy, and vascular intervention were adopted; for AIS patients within 9 h of onset, mechanical thrombectomy was performed under imaging guidance.

### 2.5 Follow‐up

All AIS patients were followed up regularly until loss to follow‐up, death, or up to 36 months. The final date of follow‐up was January 31, 2021. During 36‐month follow‐up, disease recurrence and patient's death were recorded for the further analysis.

### 2.6 Statistics

Graphics were constructed using GraphPad Prism V.8.1.1 (GraphPad Software Inc.) and R V.4.0.5 (available at: https://www.r‐project.org/), and statistical analyses were completed using SPSS V.24.0 (IBM). Difference of JKAP between AIS patients and controls was compared using Wilcoxon rank sum test, and the abilities of JKAP in distinguishing AIS patients from controls, recurrence patients form non‐recurrence patients, and death patients form survivors were evaluated using receiver operating characteristic (ROC) curves. Among AIS patients, correlation of JKAP with NIHSS score, cytokines, and adhesions molecules was determined using Spearman's rank correlation test. Correlations between JKAP and underlying diseases were analyzed using Wilcoxon rank sum test. Comparisons of JKAP between the patients with recurrence and without recurrence as well as between the dead patients and the survivors were analyzed using Wilcoxon rank sum test. Multivariate Cox's regression analysis with forward stepwise method was conducted to investigate the potential factors affecting recurrence risk and death risk in AIS patients. *p* < 0.05 was considered statistically significant.

## 3 RESULTS

### 3.1 Characteristics of AIS patients

The mean age of AIS patients was 65.8 ± 9.6 years, with 41 (33.6%) females and 81 (66.4%) males (Table 1). In terms of underlying diseases, 101 (82.8%), 59 (48.4%), 54 (44.3%), 29 (23.8%), and 25 (20.5%) AIS patients suffered hypertension, hyperlipidemia, hyperuricemia, diabetes mellitus, and chronic kidney disease, correspondingly. Besides, median NIHSS score of AIS patients was 8.0 (interquartile range (IQR): 4.0–12.0). Additionally, the detailed characteristics of AIS patients are listed in Table 1. Moreover, the clinical characteristics of the controls were listed in Table S1.

**TABLE 1:** Characteristics of AIS patients

Items	AIS patients (*N* = 122)
Demographics	
Age (years), mean ± SD	65.8 ± 9.6
Gender, *n* (%)	
Female	41 (33.6)
Male	81 (66.4)
BMI (kg/m^2^), mean ± SD	24.2 ± 2.5
History of smoke, *n* (%)	61 (50.0)
Underlying diseases	
Hypertension, *n* (%)	101 (82.8)
Hyperlipidemia, *n* (%)	59 (48.4)
Hyperuricemia, *n* (%)	54 (44.3)
Diabetes mellitus, *n* (%)	29 (23.8)
Chronic kidney disease, *n* (%)	25 (20.5)
Disease features	
No. of risk factors, *n* (%)	
1	2 (1.6)
2	61 (50.0)
3	36 (29.5)
4	19 (15.6)
5	3 (2.5)
6	1 (0.8)
NIHSS score, median (IQR)	8.0 (4.0–12.0)
Inflammatory cytokines	
IFN‐γ (pg/ml), median (IQR)	1.6 (0.8–2.3)
IL‐4 (pg/ml), median (IQR)	14.8 (12.3–20.5)
IL‐17 (pg/ml), median (IQR)	36.2 (29.0–47.2)
TNF‐α (pg/ml), median (IQR)	212.3 (161.4–299.5)
Adhesion molecule	
ICAM‐1 (ng/ml), median (IQR)	91.8 (57.3–143.0)
VCAM‐1 (ng/ml), median (IQR)	484.0 (382.1–667.3)

Abbreviations: AIS, acute ischemic stroke; BMI, body mass index; ICAM‐1, intercellular cell adhesion molecule‐1; IFN‐γ, interferon gamma; IL‐17, interleukin‐17; IL‐4, interleukin‐4; IQR, interquartile range; NIHSS, National Institute Health of Stroke Scale;SD, standard deviation; TNF‐α, tumor necrosis factor alpha; VCAM‐1, vascular cell adhesion molecule‐1.

### 3.2 JKAP level in AIS patients and controls

**FIGURE 1:**
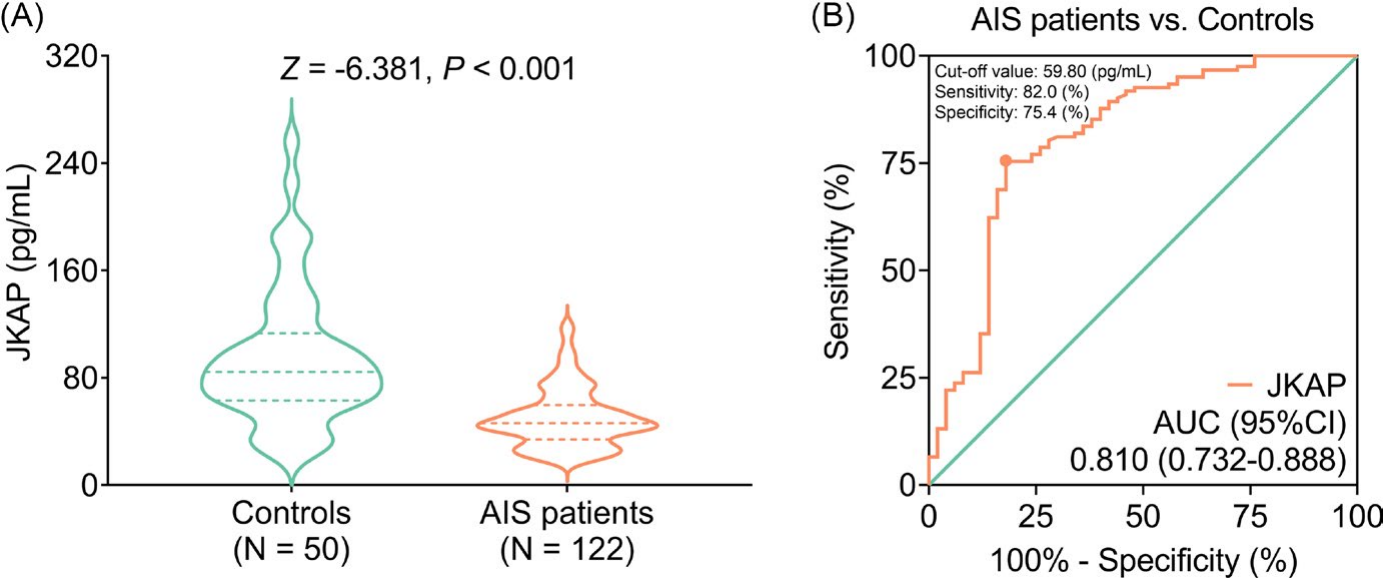
JKAP was declined in AIS patients and it could identify AIS risk. JKAP level in AIS patients and controls presented by violin graph (A). ROC curve of JKAP in distinguishing AIS patients from controls (B)

JKAP level was decreased in AIS patients (46.350 [IQR: 34.250–59.875] pg/ml) compared with controls (84.500 [IQR: 63.175–113.275] pg/ml; *p* < 0.001, Figure 1A). Moreover, further ROC curve exhibited that JKAP could work as a susceptibility biomarker in differentiating AIS patients from controls (area under curve [AUC]: 0.810, 95% confidence interval [CI]: 0.732–0.888) with the best cutoff value of 59.80 pg/ml (Figure 1B).

Furthermore, JKAP could differentiate recurrence patients from non‐recurrence patients (AUC: 0.642, 95% CI: 0.518–0.766) with the best cutoff value of 34.50 pg/ml (Figure S1A); however, it could distinguish death patients from survivors to some extent (AUC: 0.620, 95% CI: 0.426–0.813) with the best cutoff value of 34.50 pg/ml (Figure S1B).

### 3.3 Correlation of JKAP with NIHSS score, Th1/2/17 cell cytokines, adhesion molecules, and underlying diseases in AIS patients

**FIGURE 2:**
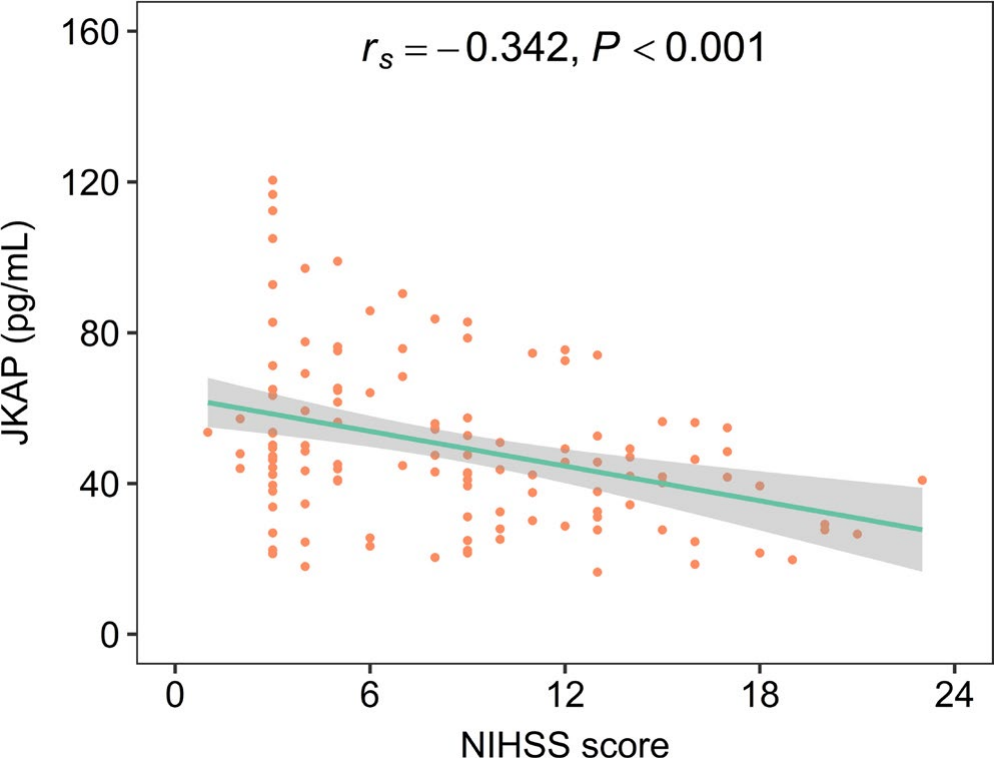
JKAP was negatively related to NIHSS score in AIS patients

JKAP was negatively linked with NIHSS score in AIS patients (*r_s_* = −0.342, *p* < 0.001, Figure 2), which indicated that JKAP was negatively correlated with overall disease severity in AIS patients.

**FIGURE 3:**
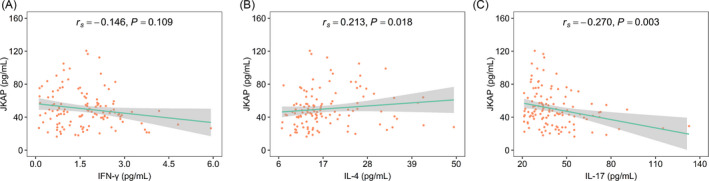
Increased JKAP linked with elevated IL‐4 and decreased IL‐17 in AIS patients. Correlation of JKAP with IFN‐γ (A), IL‐4 (B), and IL‐17 (C) in AIS patients

Moreover, JKAP was positively related to IL‐4 (*r_s_* = 0.213, *p* = 0.018), but negatively associated with IL‐17 (*r_s_* = −0.270, *p* = 0.003) in AIS patients; besides, JKAP was not correlated with IFN‐γ (*r_s_* = −0.146, *p* = 0.109) (Figure 3A–C). In brief, JKAP might be linked with Th2 and Th17 cells, but not linked with Th1 cells in AIS patients.

**FIGURE 4:**
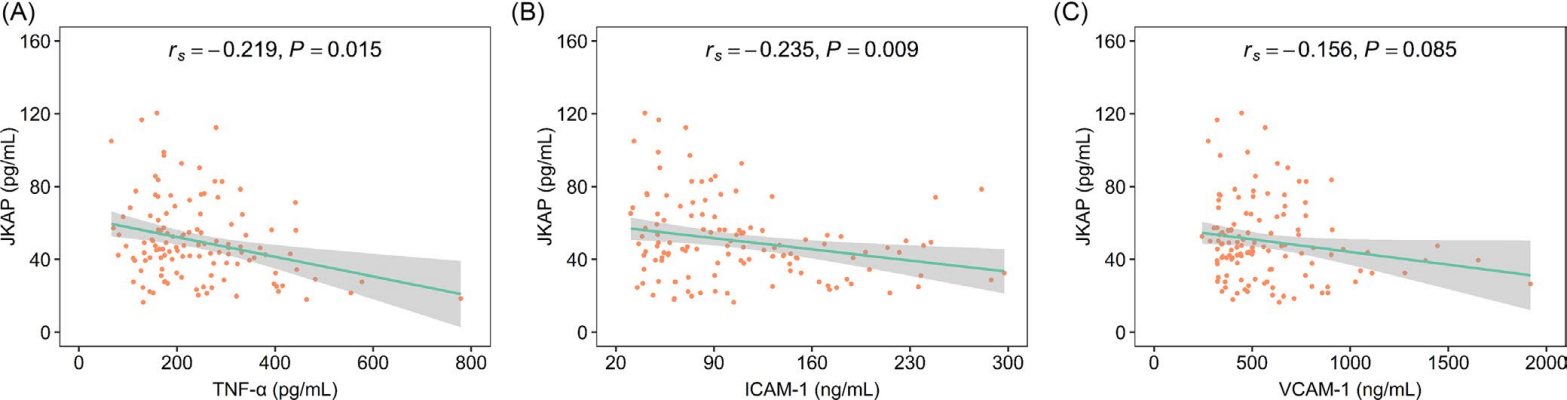
JKAP negatively associated with TNF‐α and ICAM‐1 in AIS patients. Association of JKAP with TNF‐α (A), ICAM‐1 (B), and VCAM‐1 (C) in AIS patients

Furthermore, elevated JKAP was associated with declined TNF‐α (*r_s_* = −0.219, *p* = 0.015) and ICAM‐1 (*r_s_* = −0.235, *p* = 0.009), while JKAP was not related to VCAM‐1 (*r_s_* = −0.156, *p* = 0.085) in AIS patients (Figure 4A–C). Additionally, JKAP was not correlated with underlying diseases such as hypertension, hyperlipidemia, hyperuricemia, diabetes mellitus, or chronic kidney disease in AIS patients (all *p* > 0.050) (Table S2).

### 3.4 Correlation of JKAP with recurrence and death risk in AIS patients

**FIGURE 5:**
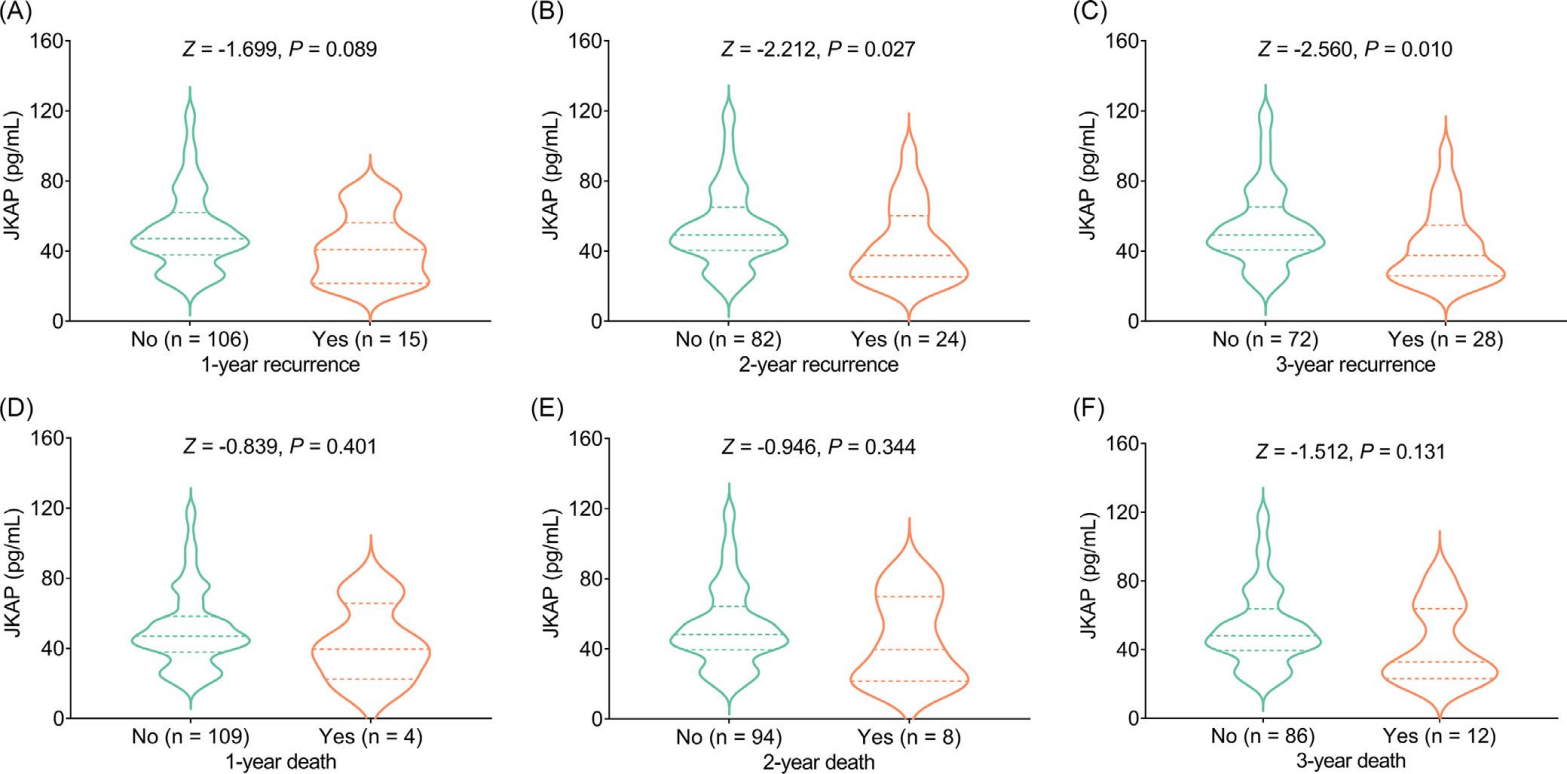
Declined JKAP correlated with 2‐year and 3‐year recurrence in AIS patients. Correlation of JKAP with 1‐year (A), 2‐year (B), and 3‐year (C) recurrence in AIS patients. Association of JKAP with 1‐year (D), 2‐year (E), and 3‐year (F) death in AIS patients. All these results were presented by violin graphs; in detail, the width of violin graphs represented the distribution of data, besides, the three lines (from top to bottom) represented the value of 3/4 quartile, the median, and 1/4 quartile, respectively

JKAP was not correlated with 1‐year recurrence in AIS patients (*Z* = −1.699, *p* = 0.089, Figure 5A). While decreased JKAP was linked with 2‐year recurrence (*Z* = −2.212, *p* = 0.027) and 3‐year recurrence (*Z* = −2.560, *p* = 0.010) in AIS patients (Figure 5B–C).

Furthermore, JKAP was not associated with 1‐year death (*Z* = −0.839, *p* = 0.401), 2‐year death (*Z* = −0.946, *p* = 0.344), or 3‐year death (*Z* = −1.512, *p* = 0.131) in AIS patients (Figure 5D–F).

In order to further verify whether JKAP was independent factor of recurrence risk and death risk of AIS, the multivariate Cox's regression analysis was conducted, which exhibited that higher JKAP was linked with declined recurrence risk in AIS patients (hazard ratio (HR): 0.969, 95% CI: 0.946–0.993, *p* = 0.011), but it was not related to death risk (HR: 0.978, 95% CI: 0.945–1.011, *p* = 0.190; Table S3). Additionally, higher age (HR: 1.074 95% CI: 1.031–1.118, *p* = 0.001), higher BMI (HR: 1.362, 95% CI: 1.130–1.641, *p* = 0.001), hyperlipidemia (vs. no) (HR: 4.836, 95% CI: 1.957–11.952, *p* = 0.001), and diabetes mellitus (vs. no) (HR: 4.283, 95% CI: 1.915–9.578, *p* < 0.001) were associated with elevated recurrence risk; also, diabetes mellitus (vs. no) (HR: 3.661, 95% CI: 1.126–11.905, *p* = 0.031) was related to increased death risk in AIS patients.

## 4 DISCUSSION

JKAP is a dual‐specificity phosphatase (DUSP), which inactivates MAP kinases (including c‐Jun N‐terminal kinase (JNK), extracellular signal‐regulated kinases (ERK), and p38 kinases) via dephosphorylating tyrosine as well as serine/threonine.^11^, ^14^, ^21^, ^22^ As MAP kinases participate in moderating immune and inflammation responses, many studies focus on the clinical role of JKAP in several inflammatory diseases (including inflammatory bowel disease, sepsis, etc.).^23^, ^24^, ^25^ For instance, one study indicates that JKAP suppresses the differentiation of T cells into Th17 cells in inflammatory bowel disease patients.^24^ Another study exhibits that JKAP is negatively correlated with Th1 and Th17 cell proportion in sepsis patients.^25^ However, related studies conducted on AIS patients are limited. In this study, upregulated JKAP was associated with increased IL‐4 and decreased IL‐17 in AIS patients, which reflected that JKAP might be positively related to Th2 cells, but negatively linked with Th17 cells. Possible explanations could be that (i) Increased JKAP inhibited the differentiation of CD4^+^ T cells into Th17 cells via inactivating lck.^26^ Thus, JKAP was negatively related to Th17 cells and its secreted cytokine (IL‐17) in AIS patients. (ii) JKAP was negatively correlated with inflammation level in AIS patients; meanwhile, Th2 cells could secrete anti‐inflammatory cytokines (such as IL‐10 and IL‐4, etc.).^27^, ^28^ As a result, JKAP was positively associated with Th2 cells and its secreted cytokine (IL‐4) in AIS patients. Notably, IFN‐γ, IL‐4, and IL‐17 were the main secreted cytokines of Th1/Th2/Th17 cells; additionally, the detection of Th cell proportion should be performed immediately, which was difficult to realize. Hence, we chose IFN‐γ, IL‐4, and IL‐17 to represent Th1/2/17 cells.

Several evidences illustrate that JKAP involves in the inflammation process in Crohn's disease, rheumatoid arthritis, and other inflammation‐mediated diseases.^14^, ^27^ For instance, one study shows that downregulated JKAP level is related to exacerbated inflammation level in Crohn's disease.^14^ Besides, it is also reported that JKAP limits T cells overproducing ICAM‐1 and VCAM‐1 in the kidney of systemic lupus erythematosus patients.^29^ Whereas, the correlation of JKAP with inflammatory status and adhesion molecules in AIS patients has not been studied yet. The current study found that JKAP was negatively correlated with TNF‐α and ICAM‐1 in AIS patients. Probable reasons could be that (i) JKAP suppressed the activation of MAP kinases signaling pathways which ultimately promoted vascular stress‐induced inflammation.^11^, ^30^, ^31^, ^32^ Hence, JKAP was negatively associated with TNF‐α in AIS patients. (ii) TNF‐α was reported to upregulate ICAM‐1 on the surface of endothelial cells in previous studies; meanwhile, JKAP was negatively linked with TNF‐α in AIS patients.^23^, ^33^ Therefore, JKAP was negatively related to ICAM‐1 as well in AIS patients.

Apart from the abovementioned findings, this study also disclosed that JKAP was negatively linked with disease severity and recurrence but not associated with death risk in AIS patients. The possible reasons might be that (i) JKAP was negatively correlated with inflammation level in AIS patients; meanwhile, inflammation was closely linked with neuronal loss and further increases cerebral injury in AIS.^31^, ^32^ Therefore, JKAP was negatively associated with disease severity in AIS patients. (ii) Sudden onset of focal neurological deficit facilitated disease severity of AIS patients, while JKAP could ameliorate neuronal function through stimulating neuron viability.^2^, ^15^ Thus, JKAP was negatively related to disease severity in AIS patients. (iii) As mentioned above, JKAP alleviated inflammation level and disease severity in AIS patients, while recurrence patients were usually accompanied by increased inflammation level and disease severity.^6^ Consequently, JKAP was negatively associated with recurrence risk in AIS patients. (iv) The reason why JKAP was not correlated with death in AIS patients might be that the number of death events in the study was relevant small which caused a low statistical power; hence, this correlation was hard to be investigated.

Some limitations existed in the current study. Firstly, the sample size of this study was relatively small; thus, further study with a larger sample size was necessary. Secondly, this study only enrolled AIS patients; however, the clinical role of JKAP in other stroke (such as hemorrhagic stroke, etc.) patients was unknown. Thirdly, the underlying mechanisms of JKAP in regulating Th cell differentiation, neural function and adhesion molecules were necessary to be explored in further study. Fourthly, the RNA level of JKAP in AIS patients was unknown, which needed further analysis. Fifthly, the main mechanism of JKAP in AIS remained unclear and needed further investigation in both in vivo and in vitro studies. Sixthly, the Th cell proportion was necessary to detect in further studies. Seventhly, the inflammatory cytokines (IFN‐γ, IL‐4, IL‐17, and TNF‐α) and adhesion molecules (ICAM‐1 and VCAM‐1) in controls were not collected in the current study. Eighthly, the original source of JKAP in serum remained unclear, while it deserved further study. Ninthly, according to the previous study, AIS patients often faced psychological issues (such as depression), which needed more attention in the further studies.^34^

In conclusion, serum JKAP correlates with disease severity, Th2 and Th17 secreted cytokines, ICAM‐1, and recurrence in AIS patients, indicating its potency to realize the risk stratification and consequently help clinicians provide individual treatment to each AIS patient.

## CONFLICT OF INTEREST

The authors declare that they have no conflicts of interest.

## Supporting information

Fig S1

Table S1

Table S2

Table S3

## Data Availability

Data sharing not applicable to this article as no datasets were generated or analyzed during the current study.
