# Supplementary figures and images for: Clinical value of serum JKAP in acute ischemic stroke patients
Source: J Clin Lab Anal. 2022 Mar 10;36(4):e24270. doi: 10.1002/jcla.24270 (PMC8993637; doi:10.1002/jcla.24270)

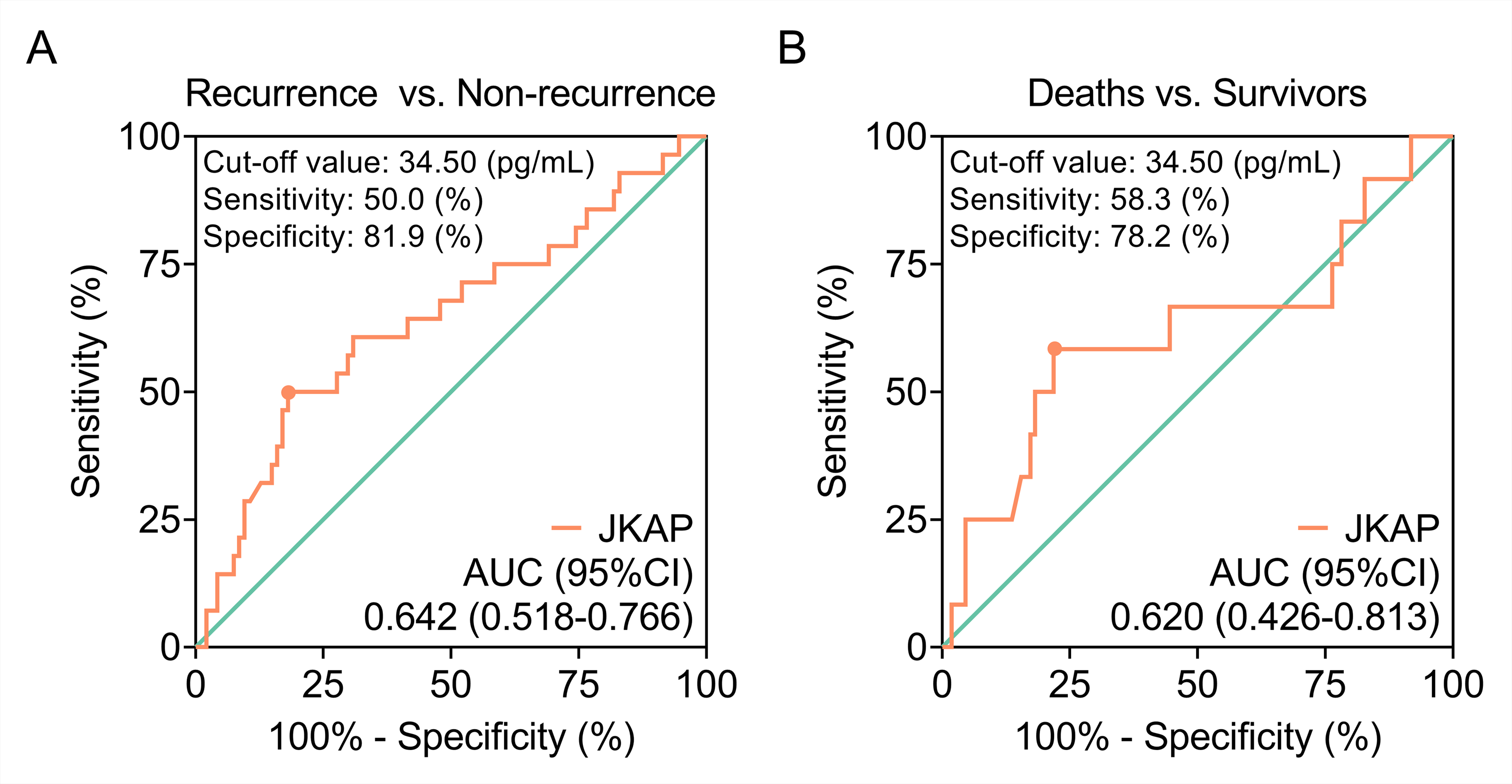

Supplement: Supplementary file 1 — Fig S1 [file JCLA-36-e24270-s004.tif]
