# Supplementary material for: Clinical value of serum JKAP in acute ischemic stroke patients
Source: J Clin Lab Anal. 2022 Mar 10;36(4):e24270. doi: 10.1002/jcla.24270 (PMC8993637; doi:10.1002/jcla.24270)
Supplement: Supplementary file 2 — Table S1 [file JCLA-36-e24270-s003.docx]

**Supplementary Table 1.** Characteristics of Controls.

| Items | Controls  (N = 50) |
| --- | --- |
| **Demographics** |  |
| Age (years), mean±SD | 64.2±8.0 |
| Gender, n (%) |  |
| Female | 20 (40.0) |
| Male | 30 (60.0) |
| BMI (kg/m^2^), mean±SD | 24.0±3.2 |
| History of smoke, n (%) | 20 (40.0) |
| **Underlying diseases** |  |
| Hypertension, n (%) | 40 (80.0) |
| Hyperlipidemia, n (%) | 26 (52.0) |
| Hyperuricemia, n (%) | 11 (22.0) |
| Diabetes mellitus, n (%) | 8 (16.0) |
| Chronic kidney disease, n (%) | 9 (18.0) |
| **Disease features** |  |
| No. of risk factors, n (%) |  |
| 2 | 38 (76.0) |
| 3 | 10 (20.0) |
| 4 | 2 (4.0) |

SD, standard deviation; BMI, body mass index; IQR, interquartile range.
