# Supplementary material for: Clinical value of serum JKAP in acute ischemic stroke patients
Source: J Clin Lab Anal. 2022 Mar 10;36(4):e24270. doi: 10.1002/jcla.24270 (PMC8993637; doi:10.1002/jcla.24270)
Supplement: Supplementary file 3 — Table S2 [file JCLA-36-e24270-s002.docx]

**Supplementary Table 2.** Correlation of JKAP with underlying diseases in AIS patients.

| Items | JKAP (pg/mL), median (IQR) | Statistic (*Z*) | *P* value |
| --- | --- | --- | --- |
| Hypertension |  | -1.621 | 0.105 |
| No | 50.2 (41.0-73.3) |  |  |
| Yes | 44.8 (33.2-56.4) |  |  |
| Hyperlipidemia |  | -0.963 | 0.335 |
| No | 47.3 (37.6-68.4) |  |  |
| Yes | 45.7 (31.1-56.3) |  |  |
| Hyperuricemia |  | -0.147 | 0.883 |
| No | 46.0 (37.7-56.1) |  |  |
| Yes | 47.2 (33.5-64.3) |  |  |
| Diabetes mellitus |  | -0.153 | 0.878 |
| No | 46.4 (33.2-61.4) |  |  |
| Yes | 44.8 (36.9-57.6) |  |  |
| Chronic kidney disease |  | -0.362 | 0.718 |
| No | 46.7 (37.8-58.3) |  |  |
| Yes | 43.1 (28.2-69.0) |  |  |

JKAP, JNK pathway-associated phosphatase; AIS, acute ischemic stroke; IQR, interquartile range.
