# Supplementary material for: Clinical value of serum JKAP in acute ischemic stroke patients
Source: J Clin Lab Anal. 2022 Mar 10;36(4):e24270. doi: 10.1002/jcla.24270 (PMC8993637; doi:10.1002/jcla.24270)
Supplement: Supplementary file 4 — Table S3 [file JCLA-36-e24270-s001.docx]

**Supplementary Table 3.** Factors affecting risk of recurrence and death by multivariate Cox's proportional hazards regression analysis.

| Items | Recurrence | | | | Death | | | |
| --- | --- | --- | --- | --- | --- | --- | --- | --- |
|  | *P* value | HR | 95%CI | | *P* value | HR | 95%CI | |
|  |  |  | Lower | Higher |  |  | Lower | Higher |
| Higher JKAP | 0.011 | 0.969 | 0.946 | 0.993 | 0.190 | 0.978 | 0.945 | 1.011 |
| Higher Age | 0.001 | 1.074 | 1.031 | 1.118 | 0.196 | 1.041 | 0.979 | 1.107 |
| Higher BMI | 0.001 | 1.362 | 1.130 | 1.641 | 0.139 | 1.211 | 0.940 | 1.562 |
| Hyperlipidemia (yes vs. no) | 0.001 | 4.836 | 1.957 | 11.952 | 0.060 | 3.606 | 0.949 | 13.712 |
| Diabetes mellitus (yes vs. no) | <0.001 | 4.283 | 1.915 | 9.578 | 0.031 | 3.661 | 1.126 | 11.905 |

HR, hazard ratio; CI, confidence interval; JKAP, JNK pathway-associated phosphatase; BMI, body mass index.
